# Midbrain and pons MRI shape analysis and its clinical and CSF correlates in degenerative parkinsonisms: a pilot study

**DOI:** 10.1007/s00330-023-09435-0

**Published:** 2023-02-11

**Authors:** C. Painous, S. Pascual-Diaz, E. Muñoz-Moreno, V. Sánchez, JC. Pariente, A. Prats-Galino, M. Soto, M. Fernández, A. Pérez-Soriano, A. Camara, E. Muñoz, F. Valldeoriola, N. Caballol, C. Pont-Sunyer, N. Martin, M. Basora, M. Tio, J. Rios, MJ. Martí, N. Bargalló, Y. Compta

**Affiliations:** 1 Parkinson’s Disease & Movement Disorders Unit, Parkinson’s Disease and Other Degenerative Movement Disorders Team, Neurology Service, Hospital Clínic de Barcelona, IDIBAPS, CIBERNED (CB06/05/0018-ISCIII), ERN-RND, Institut Clínic de Neurociències (UBNeuro), Department of Medicine, School of Medicine, Universitat de Barcelona, Catalonia Barcelona, Spain; 2 Lab of Parkinson Disease and Other Neurodegenerative Movement Disorders, Institut d’Investigacions Biomèdiques August Pi I Sunyer (IDIBAPS), Institut de Neurociències, Hospital Clínic de Barcelona, Institut de Neurociències (UBNeuro), Universitat de Barcelona, Catalonia Barcelona, Spain; 3 Magnetic Resonance Imaging Core Facility, Institut d’Investigacions Biomèdiques August Pi I Sunyer (IDIBAPS), Barcelona, Spain; 4 Laboratory of Surgical Neuroanatomy (LSNA), Universitat de Barcelona, Barcelona, Spain; 5 Centre de Diagnostic Per La Imatge (CDIC), Hospital Clinic, Barcelona, Spain; 6 Institut d’Investigacions Biomèdiques August Pi I Sunyer (IDIBAPS), Barcelona, Spain; 7 UParkinson Centro Médico Teknon, Grupo Hospitalario Quirón Salud, Barcelona, Spain; 8 Department of Neurology, Hospital Sant Joan Despí Moisès Broggi and Hospital General de L’Hospitalet, Consorci Sanitari Integral, Barcelona, Spain; 9 Neurology Unit, Hospital General de Granollers, Universitat Internacional de Catalunya, Barcelona, Spain; 10 Department of Anaesthesiology, Hospital Clinic, Barcelona, Spain; 11 Medical Statistics Core Facility, IDIBAPS & Biostatistics Unit, Faculty of Medicine, Universitat Autònoma de Barcelona, Barcelona, Catalonia Spain; 12 Neuroradiology Service, Hospital Clínic de Barcelona, 170 Villarroel Street, 08036 Barcelona, Spain

**Keywords:** Multiple system atrophy, Progressive supranuclear palsy, Shape analysis, Neurofilament protein, Parkinsonian disorders

## Abstract

**Objectives:**

To conduct brainstem MRI shape analysis across neurodegenerative parkinsonisms and control subjects (CS), along with its association with clinical and cerebrospinal fluid (CSF) correlates.

**Methodology:**

We collected demographic and clinical variables, performed planimetric and shape MRI analyses, and determined CSF neurofilament-light chain (NfL) levels in 84 participants: 11 CS, 12 with Parkinson’s disease (PD), 26 with multiple system atrophy (MSA), 21 with progressive supranuclear palsy (PSP), and 14 with corticobasal degeneration (CBD).

**Results:**

MSA featured the most extensive and significant brainstem shape narrowing (that is, atrophy), mostly in the pons. CBD presented local atrophy in several small areas in the pons and midbrain compared to PD and CS. PSP presented local atrophy in small areas in the posterior and upper midbrain as well as the rostral pons compared to MSA. Our findings of planimetric MRI measurements and CSF NfL levels replicated those from previous literature. Brainstem shape atrophy correlated with worse motor state in all parkinsonisms and with higher NfL levels in MSA, PSP, and PD.

**Conclusion:**

Atypical parkinsonisms present different brainstem shape patterns which correlate with clinical severity and neuronal degeneration. In MSA, shape analysis could be further explored as a potential diagnostic biomarker. By contrast, shape analysis appears to have a rather limited discriminant value in PSP.

**Key Points:**

• *Atypical parkinsonisms present different brainstem shape patterns*.

• *Shape patterns correlate with clinical severity and neuronal degeneration*.

• *In MSA, shape analysis could be further explored as a potential diagnostic biomarker*.

**Supplementary Information:**

The online version contains supplementary material available at 10.1007/s00330-023-09435-0.

## Introduction

Structural MRI has been widely studied in the differential diagnosis of neurodegenerative parkinsonisms: Parkinson’s disease (PD), multiple system atrophy (MSA), progressive supranuclear palsy (PSP), and corticobasal degeneration (CBD). Many studies have focused on the brainstem due to its frequent involvement in atypical parkinsonisms (AP: PSP, MSA, CBD). Atrophy of the midbrain in PSP [1–3] or the pons in MSA [4, 5] is a well-known example. Structural MRI brainstem measures include morphological markers (“hummingbird” sign in PSP[6]; “hot cross bun” sign in MSA [7]), quantitative measures (midbrain anterior–posterior diameter and brainstem midsagittal areas or volumes) [8], and specific ratios [9–11]. Several of these measurements can discriminate between PD, PSP, and MSA in isolation [10–12] or combined with other biomarkers [13]. However, results have been variable for morphological [14] and the antero-posterior diameter of the midbrain [8, 9].

An alternative approach is shape analysis, which detects local narrowing in specific regions of complex structures, as opposed to conventional volumetric analysis only reporting changes in the overall volume [15]. This technique has been used in neurodegenerative parkinsonisms to study local atrophy patterns in the basal ganglia, the thalamus, and the hipoccampus [16–21], but not the brainstem, nor its CSF biomarkers correlates either, to the best of our knowledge.

With the hypothesis that brainstem MRI shape analysis might differentiate neurodegenerative parkinsonisms, we aimed at characterizing and comparing brainstem shape changes across these conditions, as well as analyzing the clinical and biological correlates of shape changes. As secondary goals, we intended to replicate prior findings of cerebrospinal fluid (CSF) levels of neurofilament-light chain (NfL) and automatic measures of the pons to midbrain ratio (PM) ratio.

## Methods

### Design

This is a cross-sectional study of patients recruited between 2015 and 2020 at the Parkinson’s Disease & Movement Disorders Unit of the Hospital Clinic in Barcelona as part of different research projects implying the availability of both high-field MRI and CSF samples for almost each participant.

### Participants

There was not a formal sample size calculation, but rather a post hoc analysis of the aforementioned projects considering that sample size was in the range of prior published studies on this topic, along with the uniqueness of our cohort due to having available both MRI and CSF data in almost all patients (unlike previous published literature where MRI shape analyses were not correlated with CSF findings) [16–21]. Hence, we included 84 subjects from two prospective biomarkers studies (Supplementary Fig. 1) conducted at our unit with 32 participants previously described in two reports on CSF cytokine levels and longitudinal clinical progression in MSA, respectively, thus not overlapping with the current study [22, 23]. All diseased-participants fulfilled the “probable” (or “clinically established” in PD) category of their respective diagnostic criteria [24–27]. CS were individuals over > 55 years, undergoing intradural anesthesia for knee surgery who, as per thorough clinical history and examination (including a Montreal Cognitive Assessment [28] (MoCa) score ≥ 26), did not have any neurological or psychiatric condition. Vascular or drug-induced parkinsonism, large vascular MRI abnormalities, and Alzheimer’s disease CSF biochemical profile in patients with corticobasal syndrome [29] were exclusion criteria. The study received approval by the Ethics Committee. All participants signed informed consent.

### Clinical procedures

Movement disorders specialized neurologists (Y.C., C.P., A.P.) collected the following demographic and clinical variables of all participants: age at disease onset, sex and age and disease duration at the time of the study procedures. Cognition was assessed by means of the MoCa [24] (except Mini Mental test (MMSE) [30] in MSA as part of an independent protocol [23]). Motor assessments were based on the Unified Multiple System Atrophy Rating Scale (UMSARS) [31] in MSA patients, the PSP Rating Scale (PSPRS) [32] in PSP, and the subscale of the Unified Parkinson’s Disease Rating Scale (UPDRS part III) [33] in all subjects except in MSA subjects. Hoehn and Yahr classification (HY) [34] was obtained for all the participants. Disability assessment was based on the Schwab and England Activities of Daily Living (SEADL) scale [35].

### CSF collection, storage, and analyses

CSF samples were obtained via lumbar puncture at the L3–L4 level with a 22-gauge needle, between 8 and 10 a.m. The first 2 mL was used for routine studies. CSF was processed within 30 min of collection, centrifuged at 2000 rcf and 4 °C for 10 min, and stored at − 80 °C [36]. CSF NfL levels were measured with a commercial ELISA kit (Umandiagnostics, Sweden). The samples were run together with blank (sample diluent), the (prepared) calibrator solutions, and the appropriate control always in duplicate (a single concentration value in pg/mL was calculated as the mean of the duplicates; all with a variation coefficient < 20%).

### MRI acquisition

MRI was performed within 2 months of lumbar puncture. MRI was acquired with a 3-T Prisma Siemens scanner, including sagittal T1-weighted volumes acquired with 3-dimensional magnetization prepared rapid gradient echo (3D-MPRAGE) sequences with TR = 2.4 s, TE = 2.22 ms, FlipAngle = 8; and isometric voxel size of 0.8 × 0.8 × 0.8mm^3^. Regarding the participants coming from previous studies (see above), 23 subjects were acquired with TE = 2.98 ms, TR = 2.98 s, and voxel size 1 × 1 × 1 mm^3^. The T1-weighted MRI acquisition parameters for the other 10 subjects ranged from TE = 2.17 to 6.30 ms, TR = 14.40 ms to 2.4 s, and voxel sizes between 0.94 × 0.94 × 0.94 and 1.2 × 1.05 × 1.05 mm^3^. Details on the number of acquisitions with each protocol per group can be found in Supplementary table 1. Supplementary Fig. 2 shows the compatibility between acquisition protocols in planimetry metrics and shape analyses.

### Morphometric MRI measurements

#### Brainstem automatic measures (Supplementary Fig. 3)

Brainstem structures were parcelled using the Brainstem Bayesian FreeSurfer module [37]. First, for each subject, the T1-weighted image was aligned with a template in the MRI to identify the midsagittal plane. To account for variability in the alignment, 10 slices around the central sagittal slice were evaluated to identify the midsagittal slice, which was defined as the one containing the smaller midbrain area. To automatically assess midsagittal midbrain area (*M*_A_) and midsagittal pontine area (*P*_A_), the number of voxels segmented as midbrain and pons in the midsagittal slice were counted and multiplied by voxel size and PM was calculated. To validate the results of the automated measurements, the *M*_A_ and *P*_A_ measurements were replicated manually (Supplementary Fig. 4), as previously described [38], by two independent anatomical experts (“Manual 1” and “Manual 2”) blinded to clinical information. After the validation of the automatic method, the remaining stages were automatically performed.

#### Shape analysis

Pons and midbrain regions obtained from the FreeSurfer Brainstem Bayesian parcellation module were merged and modelled using Spherical Harmonics Point Distribution Models (SPHARM-PDM) obtained from the SlicerSALT software (http://salt.slicer.org/) [15, 39]. For each subject, a brainstem surface containing 1002 vertices was generated and centered in a common space. Morphological vertex-level group differences were analyzed using a multivariate functional shape data analysis (MFSDA) [40], including age as a covariate, due to significantly younger age in MSA vs. the other subgroups (see results), in keeping with known age at onset of these conditions [41]. Multiple comparisons were controlled by family-wise error (FWE) (corrected significance threshold ≤ 0.05). Finally, the distance between each vertex in the subject mesh and the corresponding vertex in the control average mesh was correlated with CSF NfL, UPDRS, PSPRS, UMSARS, and SEADL by Spearman partial correlation covaried for age. We interpreted narrowing as atrophy and enlargement as lack of atrophy or compensatory enlargement in a region near an atrophic area.

#### Statistics

Sample size was defined on a pragmatic basis considering previous studies and the rarity of atypical parkinsonisms. Qualitative variables are presented as frequencies and were compared by means of Fisher’s exact test. Quantitative data are presented as median/interquartile range (IQR) and were compared using Kruskal–Wallis test or Mann Whitney’s *U*-test, as appropriate. CSF and morphometric quantitative MRI biomarkers were compared between diagnostic groups using non-parametric analysis of covariance with age as covariate. To study the influence of CSF and MRI biomarkers (independent variables) on clinical variables (dependent variables) in parkinsonian disorders, we first transformed into ranges the independent variables and then applied multiple linear regression limiting covariables to age to minimize the risk of overfitting. For statistical purposes, HY was converted to a binary variable as HYbin: I-II vs. III-V.

To verify intra-rater and automatic-to-manual agreement, intraclass correlation coefficients (ICC) of agreement and consistency were calculated. These were considered as poor (ICC < 0.40), fair (0.40–0.59), good (0.60–0.74), and excellent (0.75–1.00) [42].

Statistical tests were two-tailed, with significance set at ≤ 0.05, corrected for multiple comparisons by false-discovery rate (FDR) [43] (except for shape analysis, FWE-corrected; see above). Subject missing values in a particular field were not included in the analysis for that particular outcome. Data analysis was carried out using Stata 16.0 (Stata Corp) for Windows and IBM SPSS statistics software version 24.0 (Armonk, NY:IBM Corp).

## Results

### Demographic and clinical data

We included 21 patients with PSP (14 PSP-Richardson’s syndrome (PSP-RS), 6 PSP parkinsonism (PSP-P), and 1 PSP-progressive gait freezing (PSP-PGF)), 14 CBD, 26 MSA (18 MSA-parkinsonian (MSA-P) and 8 MSA-cerebellar (MSA-C)), 11 PD, and 12 control subjects. The clinical diagnosis was neuropathologically confirmed in three participants (PD, CBD, and MSA; 1 each). Demographic and clinical data are shown in Table 1. There were no significant differences in disease duration, sex, and age between groups, except for MSA patients who were younger than PSP, PD, and CBD. There were no significant differences in UPDRS, HY, MoCa, and SEADL across the parkinsonian disorders. MSA patients presented a median of 28 points in the MMSE (normal value ≥ 24).

**Table 1:** Comparison of demographic and clinical data across the different study groups

	PSP (*n* = 21)	CBD (*n* = 14)	MSA (*n* = 26)	PD (*n* = 11)	CS (*n* = 12)	*p* value*
Age†	74.5 [70.0–77.4]	69.8 [67.7–74.7]	62.3 [55.1–66.8]	74.7 [64.9–77.1]	71.8 [62.7–73.7]	All groups: 0.001; PSP/CBD: 0.315; PSP/MSA: < 0.001; PSP/PD: 0.768; PSP/CS: 0.162; CBD/MSA: 0.001; CBD/PD: 0.759; CBD/CS: 0.872; MSA/PD: 0.030; MSA/CS: 0.058; PD/CS: 0.457
Gender (females)	7 (33.3)	11 (78.6)	11 (42.3)	6 (54.5)	5 (41.7)	All groups: 0.218; PSP/CBD: 0.072; PSP/MSA: 0.758; PSP/PD: 0.469; PSP/CS: 0.818; CBD/MSA: 0.140; CBD/PD: 0.594; CBD/CS: 0.218; MSA/PD: 0.818; MSA/CS: 1; PD/CS: 0.810
Disease duration†	5.4 [3.1–7.3]	5.1 [3.7–6.8]	3.8 [2.5–6.4]	7.8 [1.2–13.5]	-	All groups (except CS): 0.759; PSP/CBD: 1; PSP/MSA: 0.457; PSP/PD: 0.832; CBD/MSA: 0.558; CBD/PD: 0.768; MSA/PD: 0.540;
UPDRS	37 [26–44]	48 [42–65]	-	29 [23–43]	-	PSP/CBD/PD: 0.191; PSP/CBD: 0.118; PSP/PD: 0.697; CBD/PD: 0.218
UMSARS	-	-	44 [32.5–67.5]	-	-	
PSPRS	35 [30–43]	-	-	-	-	
HY_bin_ (HY < 3)	4 (19.05)	5 (35.71)	8 (30.77)	6 (54.55)		All groups (except CS): 0.440; PSP/CBD: 0.631; PSP/MSA: 0.697; PSP/PD: 0.155; CBD/MSA: 1; CBD/PD: 0.631; MSA/PD: 0.457
MoCa	19 [16–25]	19 [9.5–26.5]	-	20.5 [10–27]	26 [26–28.5]	PSP/CBD/PD: 0.997; PSP/CBD: 0.972; PSP/PD: 0.923; **PSP/CS:** 0.020; CBD/PD: 0.818; CBD/CS: 0.118; PD/CS: 0.789
MMSE	-	-	28 [26.5–30]	-	-	
Schwab and England	60 [45 – 75]	45 [40–60]	50 [40 – 75]	85 [50 – 95]	100 [97.5 – 100]	All groups (except CS): 0.138; PSP/CBD: 0.144; PSP/MSA: 0.859; PSP/PD: 0.439; PSP/CS: 0.001; CBD/MSA: 0.118; CBD/PD: 0.111; CBD/CS: 0.001; MSA/PD: 0.218; MSA/CS: 0.001; PD/CS: 0.051

Quantitative variables are presented as median [IQR] and compared between groups using Kruskal–Wallis or Mann–Whitney *U* tests as appropriate

Categorical variables are presented as absolute frequency (proportion) and compared between groups using Fisher’s exact test

^†^At the time of the lumbar puncture and MRI (expressed in years)

^*^p values results are presented FDR-corrected

Statistical significant differences between groups are marked in bold

### CSF biomarkers

These are summarized in Supplementary table 2. CSF NfL levels were significantly higher in atypical parkinsonisms vs. PD and CS.

### MRI quantitative measures

Automatic and manual measures are summarized in Supplementary table 2 and Supplementary table 3, respectively. For inter-rater agreement and consistency coefficients, see Supplementary table 4. MRI quantitative automatic measures at group level demonstrated that in PSP the *M*_A_ was significantly reduced, except when comparing with CBD, and the PM ratio was significantly increased when comparing with all the other groups. CBD patients presented significantly lower *M*_A_ compared to PD and CS, and the PM ratio was significantly lower when compared to PSP and higher when compared to MSA and PD. Finally, MSA patients presented significantly lower *P*_A_ than PSP, PD, and CS, and lower PM ratio than PSP and CBD. Significant associations of CSF and automatic MRI measures with clinical variables are summarized in Supplementary table 5.

### Shape analysis

#### Comparison of brainstem shape across parkinsonian disorders and control subjects

**Fig. 1:**
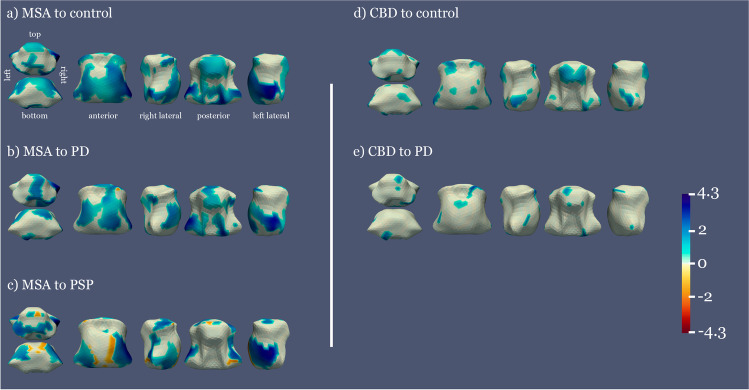
Mean distance between the average shape of each pair of groups, significant results. Color areas represent the distance between average shapes in the regions where significant differences between groups are observed (*p* < 0.05, FWE corrected). Warm to cold colormap showing areas in blue where the first group is significantly narrower than the second group and red for the opposite case. The vertical barcode represents the distance between groups in millimeters, 4.3 mm being the maximum distance found between the two groups

Areas with statistically significant shape differences are shown in Fig. 1. MSA was the group with the most extensive significant atrophy of the pons and midbrain when compared to PD and CS and mainly in the lateral inferior pons (including the middle cerebellar peduncle) when compared to PSP. CBD presented significant atrophy in several small areas in the pons and midbrain when compared to PD and CS. PSP presented significant atrophy in the upper posterior midbrain and small areas in the rostral pons when compared to MSA. For descriptive purposes, distance maps between average group shapes can be found in Supplementary Fig. 5 and information regarding the parcellation of the brainstem for the corresponding shape analysis in Supplementary Fig. 6.

#### Association of brainstem shape and clinical variables

**Fig. 2:**
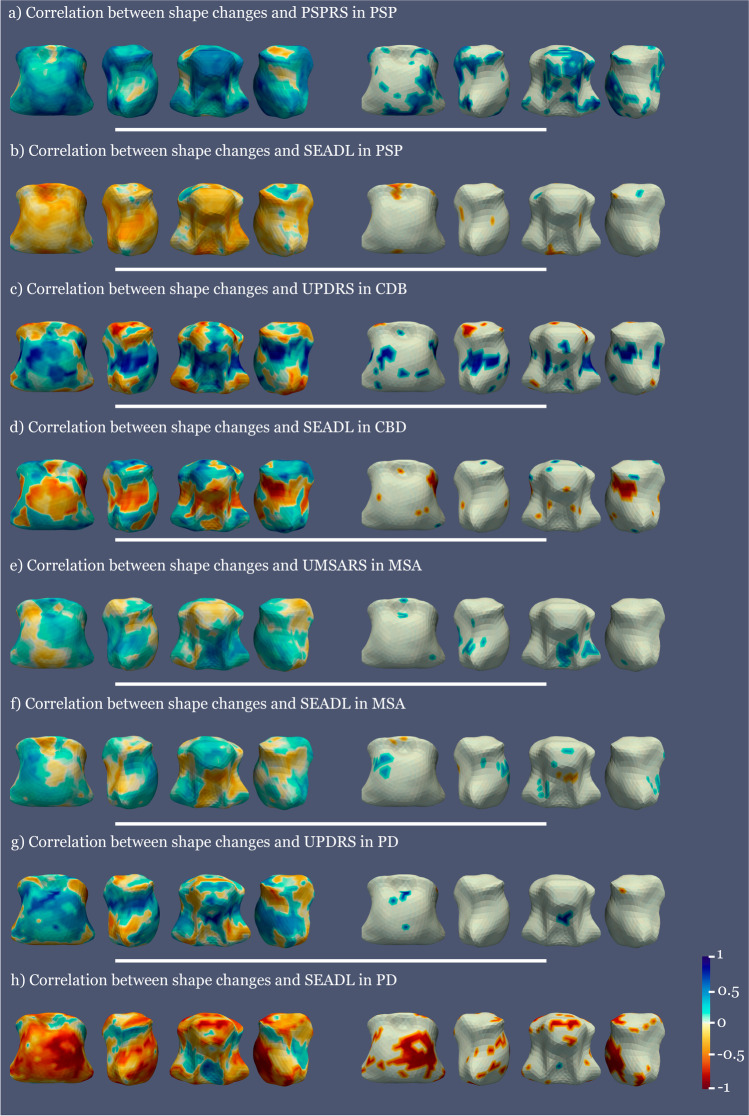
Correlation maps between clinical variables and shape atrophy in PSP, CBD, MSA, and PD compared to controls. The left column shows all the correlation values and the right column only the significant ones. Blue represents positive correlations and red negative correlations. All analyses covaried for age at inclusion

We found significant positive correlations between higher motor scales’ scores (UMSARS in MSA, PSPRS in PSP, and UPDRS in CBD and PD) and greater brainstem shape atrophy across the different parkinsonisms (Fig. 2). Specifically, in MSA, these positive correlations were present mostly in 2 small areas in the rostral upper midbrain and in a greater area in the dorsal and lateral pons. In CBD, scattered areas of positive correlations were seen in the pons. In PSP, these were present in the central posterior midbrain, the dorsal pons, the lateral midbrain, and the lateral and inferior rostral pons. In PD, positive correlations were seen in a few small areas in the rostral and dorsal pons.

We also found significant predominantly negative correlations between SEADL scores and brainstem shape alterations (that is, the more atrophy the lower SEADL scores) in CBD, PSP, and PD. Specifically, negative correlations predominated in the midbrain in CBD and PSP, and were scattered through the midbrain and the pons in PD. In MSA, there was a more mixed pattern of both negative and positive correlations (that is, atrophy or enlargement of brainstem shape was related to lower SEADL scores depending on the area) with the negative correlation surfacing in the middle posterior pons.

#### Association of brainstem shape and CSF NfL

**Fig. 3:**
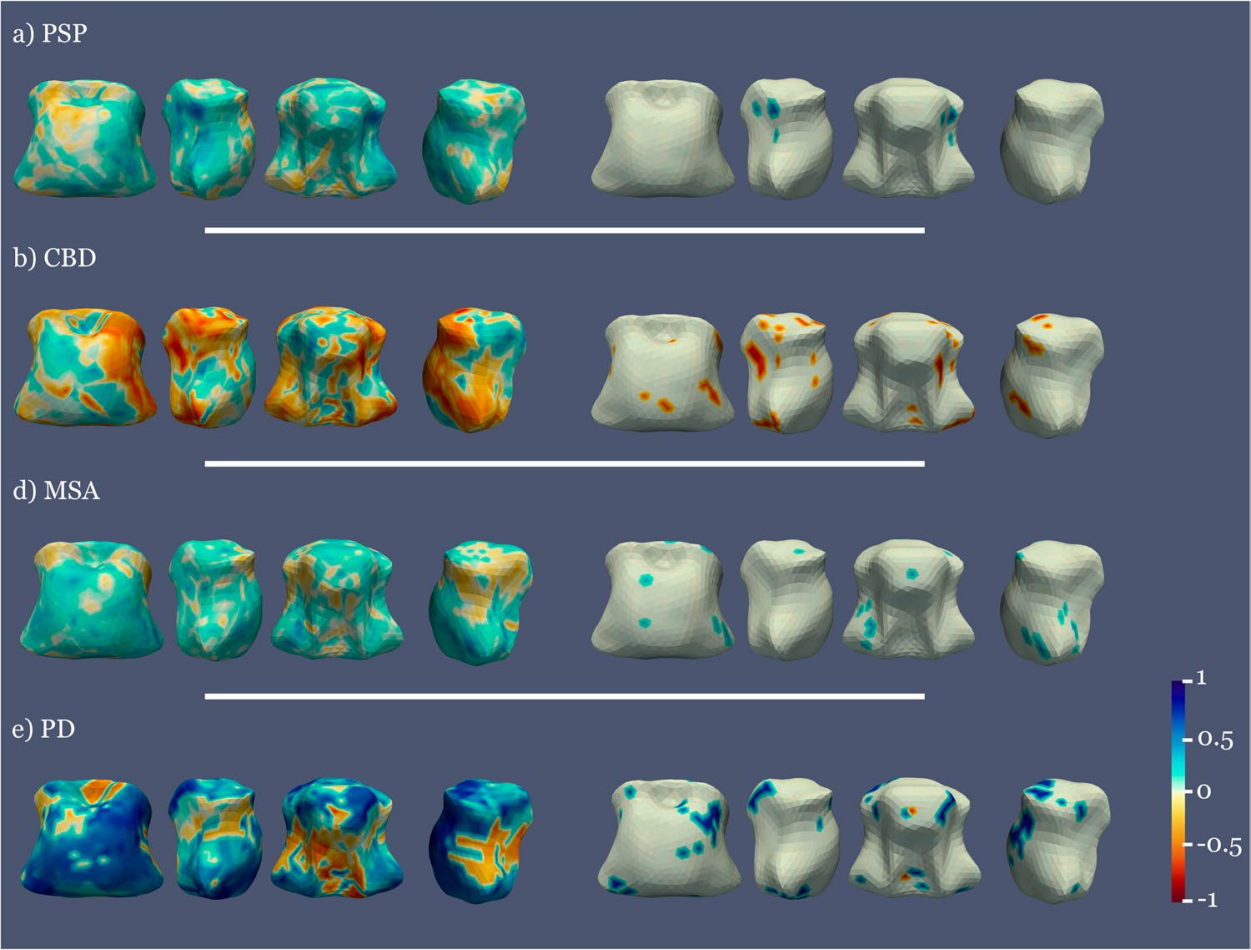
Correlation between NfL and shape atrophy in the diseased groups with respect to controls. The left column shows all the correlation values and the right column only the significant ones. Blue represents positive correlations and red negative correlations. All analyses covaried for age at inclusion

A positive correlation between CSF NfL levels and shape (the higher CSF NfL levels, the more atrophy in these areas) in PSP, MSA, and PD was seen in several small midbrain and pons areas. In CBD, significant negative correlations (enlargement of these areas related to increased NfL) were seen (Fig. 3).

## Discussion

We herein report for the first time: (1) different brainstem atrophy patterns across neurodegenerative parkinsonisms with remarkable differences in MSA; (2) local atrophy association with clinical and CSF variables across the spectrum of degenerative parkinsonisms. In addition, we have replicated previous findings of PM ratio [44, 45] and CSF NfL levels [46] in degenerative parkinsonisms.

The novelty from our study comes from brainstem shape analysis. Shape analysis has become of increasing interest in neurodegenerative diseases, as Alzheimer and Parkinson’s disease. Significant shape differences have been found between PD and control subjects in different subcortical structures, including the subthalamic nucleus [16], the globus pallidus [17], and the striatum [18, 19]. To our knowledge, studies comparing shape differences between atypical parkinsonism and healthy controls have only been performed in PSP [20, 21]. Moreover, only one study has focused on shape analysis differences between neurodegenerative parkinsonian disorders, focusing on subcortical supratentorial structures instead of the brainstem with a small sample size (5 PSP, 6 CBD, 9 PD, 12 healthy controls), and reporting significant local bilateral atrophy in the ventral anterior and ventral lateral thalamus in PSP + CBD vs. the other groups [47].

To interpret narrowing as atrophy and enlargement as inflammation or regional elastic compensation, first we compared diseased vs. control groups, then compared diseased groups, shifting from an atrophy vs. non-atrophy paradigm to an atrophy-differences one. In this vein, our results show a gradation ranging from a more extensive affectation in MSA, followed by CBD to a more limited one in PSP (Fig. 1). Shape analysis results are interpreted based on the brainstem architecture. When a global atrophy is present in the brainstem nuclei, we found significant atrophy in a larger part of the brainstem surface due to collapse of the brainstem scaffold. On the other hand, when atrophy is present in superficial structures, such as the motor tracts contained in the crus cerebri or the midbrain tectum, we obtain more localized significant atrophy surfaces. Hence, comparing MSA vs. controls, we found global surface atrophy of the pons due to atrophy of the middle cerebellar peduncles (lateral region) and an anterior–posterior collapse possibly driven by changes in pontine nuclei and transverse fibers. Midbrain surface anterior and posterior alterations (colliculi and tectum), with no significant results in the lateral regions, may also indicate an anterior–posterior collapse. Involvement of corticospinal tract in MSA [26] or of a wider region of the crus cerebri or part of the substantia nigra was also found, since atrophy of the central nuclei such as the raphe would not lead to a collapse in a structure so distal thanks to the robust architecture of the brainstem. In MSA vs. PD, local atrophy was similar but more restricted, whereas in MSA vs. PSP higher atrophy in the middle cerebellar peduncles was consistent with previous imaging and pathological knowledge [10, 48]. In the case of CBD, focal atrophy in the tectum and several small areas in the pons was observed [49].

The similar atrophy gradation of MSA and CBD vs. CS (more marked) and vs. PD (more limited) is clinically and radiologically plausible since PD is the diseased group with lesser brainstem affection [2], hence lying between APs and CS.

**Fig. 4:**
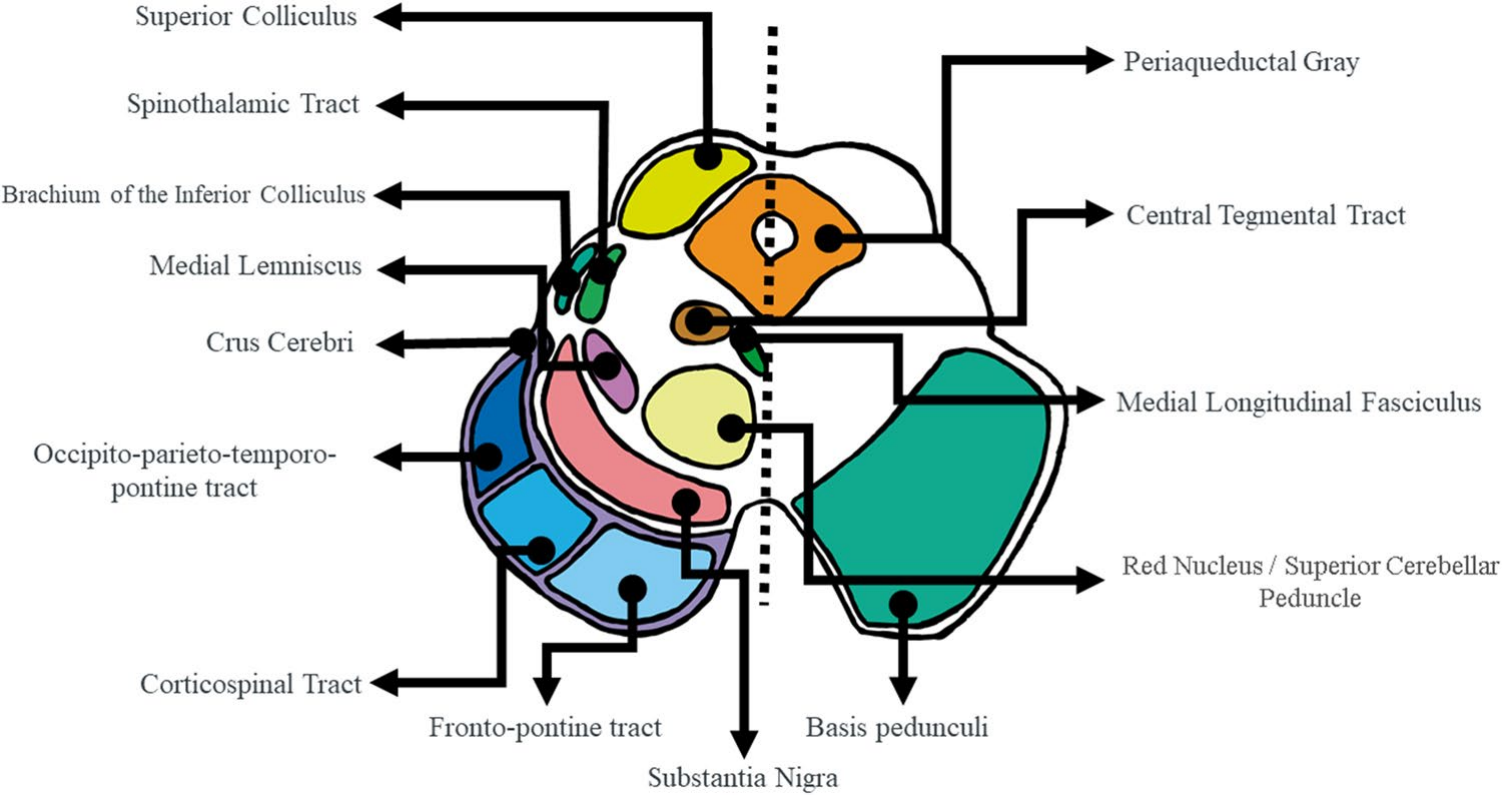
Diagram of the midbrain anatomy at the level of the superior colliculi. The diagram highlights the important contribution of the cerebral peduncles to the midbrain shape, which might turn these structures, rather spared in PSP, into “shape-preserving structures” in contrast to more large shape collapse of the pons in MSA due to more extensive pontine involvement in this condition

Greater atrophy in PSP in the superior colliculus (upper midbrain) indicates greater dento-rubro-thalamic tract and red nucleus involvement (Fig. 4) in keeping with vertical sight limitation in PSP patients, to which the superior colliculus is critical [50]. We interpret the rather restricted narrowing in PSP as follows: while in PSP atrophy is important in the whole midbrain as captured by other measures (MRPI or PM ratio), its predominance in the tectum could turn the rather spared cerebral peduncles into “shape-preserving structures” that might account for lesser ability of MRI shape analysis to detect midbrain atrophy. Alternatively, the inclusion of different PSP phenotypes, some of them with lesser burden of brainstem pathology [2, 51], might have increased the variability and reduced the significance of shape results in this group.

### Correlations with clinical and CSF variables

Greater brainstem shape atrophy was associated with worse motor state in all parkinsonisms and worse daily living function scores in CBD, PSP, and PD, while in MSA patients the correlations with SEADL were mixed: the negative correlation (the more narrowing, the lower SEADL) arose in the posterior middle pons, where tegmental pontine nucleus involvement has been correlated with severe MSA-related orthostatic hypotension, one of the most disabling MSA symptoms [52]. The positive correlations (enlargement related to lower SEADL) might be due either to a ceiling effect or to relative enlargement of certain areas in the setting of atrophy of diseased regions.

The association of greater atrophy with higher NfL levels in PSP, MSA, and PD is in keeping with the notion that CSF NfL levels indicate neuroaxonal damage. The finding of higher NfL associated with lesser atrophy in CBD is difficult to interpret. A stochastic association is unlikely due to stringent FWE correction and multivariate analyses. An alternative explanation is that it could reflect ongoing neuronal injury in areas still undergoing inflammation before atrophy [53]. In CBD, post-mortem [54] and in vivo studies [55] have demonstrated microglial activation in areas associated with tau pathology including the brainstem.

APs presented higher CSF NfL levels compared to PD and CS, but no significant differences among themselves, with increasing NfL levels significantly associated with disease severity, in agreement with previous literature [56, 57].

Shape analysis provides novel and complementary information with respect to other traditional atrophy metrics and can contribute to a better understanding of the pathological processes. First, compared to traditional indices based on planimetric measurements, shape analysis provides 3D sensitivity, that is, it is able to detect changes in all the structure, not only in the MR slice of interest. In this sense, clear differences between MSA and PSP were found by shape analysis out of the midsagittal plane. With regard to other methods quantifying atrophy as a decrease in the total volume of the anatomical region, since they provide one only measure for the whole region, they might not be sensitive to scenarios where the difference is not only the global decrease or volume but rather the specific location of the volume decrease (as shown in the comparison between MSA and PSP).

Our study is not without limitations. The different subgroups size is relatively small, yet in the range of previous published studies on MRI shape in parkinsonisms [17, 20, 21] and remarkable considering the rareness of the atypical parkinsonisms, as well as the fact that it is challenging having both MRI and CSF from the same subjects, which is unique to our study relative to prior literature [16–21]. Moreover, we have applied statistical correction for multiple comparisons and limited covariables in regression analyses to age at inclusion, thus respectively minimizing the risk of stochastic results and of overfitting. On the other hand, the fact that the sample size is small increases the risk of statistical error type II, that is, falsely rejecting the alternative hypotheses. As our study is positive with several significant findings, the potential limitation of our sample size would be that of underestimating, rather than overestimating, our findings. Neuropathological diagnosis confirmation is lacking in most cases. However, those having come to autopsy were confirmed, and for the rest strict diagnostic criteria were applied and cases with corticobasal syndrome with CSF Alzheimer-profile were excluded. The sample size was small, yet in the range of previous studies [2, 13, 58]. We considered MSA patients as a sole group but did not assess separately MSA-C and MSA-P. However, MSA-C and MSA-P share involvement of the same brain structures including the brainstem and accordingly in the diagnostic criteria the radiological findings of one variant are accepted to assist the diagnosis of the other one [26, 59]. Age differed among groups as expected since MSA usually has younger age at onset [41], but we covaried analyses for age and moreover it is unlikely that differences in age drove the results when most significant differences were obtained in the MSA (that is, the younger) group. Another limitation is the difference in T1-weighted MRI parameters as participants came from different projects. Although de novo acquisitions were acquired with the same acquisition protocol, the images from previous projects had been acquired with different parameters. The automatic methods for brainstem segmentation have shown to be robust against differences in acquisition parameters [60]. On the other hand, recent studies have shown that shape features are more robust to acquisition parameters than features related to volume or intensity [61], pointing to reliability of shape analysis even in case of differences in acquisition.

In conclusion, we have found different patterns of local brainstem atrophy across atypical parkinsonisms by means of MRI shape analyses in association with clinical and CSF indicators of disease severity. More specifically, shape analysis might be further explored as a potential MSA diagnostic biomarker. In contrast, and despite its significant clinical and CSF correlations, in PSP shape analysis appears to be of rather limited discriminant value. Our results remain preliminary and additional prospective studies of larger cohorts will help confirm or not our findings and should further assess the combination of MRI shape analysis and CSF biomarkers.

## Supplementary Information

Below is the link to the electronic supplementary material.

Supplementary file1 (DOCX 1862 KB)

## Data Availability

Data will be made available upon reasonable and justified request.

## Abbreviations

AP: Atypical parkinsonism
CBD: Corticobasal degeneration
CS: Control subjects
CSF: Cerebrospinal fluid
M_A_: Midbrain area
MFSDA: Multivariate functional shape data analysis
MRI: Magnetic resonance imaging
MSA: Multiple system atrophy
MSA-C: MSA-cerebellar
MSA-P: MSA-parkinsonian
NfL: Neurofilament-light chain
P_A_: Midsagittal pontine area
PD: Parkinson’s disease
PM: Pons to midbrain ratio
PSP: Progressive supranuclear palsy
PSP-P: PSP parkinsonism
PSP-PGF: PSP-progressive gait freezing
PSP-RS: PSP-Richardson’s syndrome

## Declarations

### Guarantor

The scientific guarantors of this publication are the corresponding authors on behalf of their institutions: Hospital Clínic de Barcelona, IDIBAPS and University of Barcelona.

### Statistics and biometry

Mr. José Ríos from the IDIBAPS Statistical Core Facilty kindly provided statistical advice for this manuscript. Dr Celia Painous and Yaroslau Compta along with Mr. Saul Pascual-Diaz and Mrs Emma Muñoz have significant statistical expertise.

### Study subjects or cohorts overlap

Out of the 84 participants, 32 were previously reported in two published studies with no overlap with the current one:

- Compta Y, Dias SP, Giraldo DM, et al. (2019) Cerebrospinal fluid cytokines in multiple system atrophy: A cross-sectional Catalan MSA registry study. Parkinsonism Relat Disord 65:3–12.
- Pérez-Soriano A, Giraldo DM, Ríos J, Muñoz E, Compta Y, Martí MJ; Catalán MSA Registry (CMSAR) (2021) Progression of Motor and Non-Motor Symptoms in Multiple System Atrophy: A Prospective Study from the Catalan-MSA Registry. J Parkinsons Dis 11(2):685–694.

### Methodology

- prospective
- cross-sectional
- observational
- multicenter study
