# Supplementary material for: Midbrain and pons MRI shape analysis and its clinical and CSF correlates in degenerative parkinsonisms: a pilot study
Source: Eur Radiol. 2023 Feb 11;33(7):4540–51. doi: 10.1007/s00330-023-09435-0 (PMC10290009; doi:10.1007/s00330-023-09435-0)
Supplement: Supplementary file 1 — Supplementary file1 (DOCX 1862 KB) [file 330_2023_9435_MOESM1_ESM.docx]

**Supplementary Fig. 1 Participant flow chart.**

We included 84 subjects from two prospective biomarkers studies. This figure shows the participant flow chart including the eligibility criteria.

**
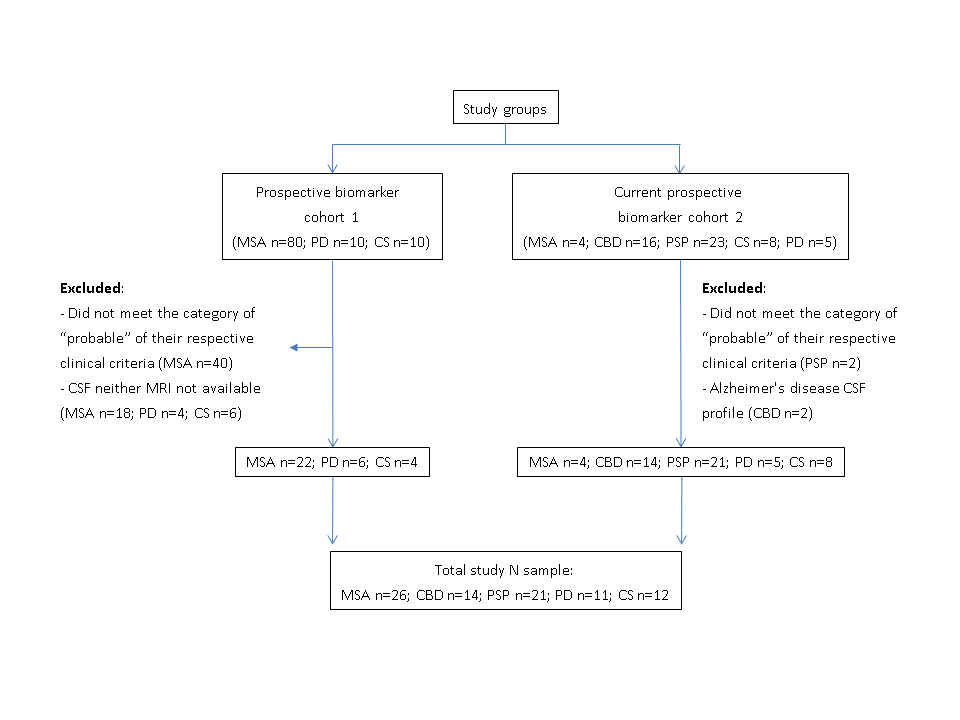
**

**Supplementary Fig.2**

Compatibility between acquisition protocols in planimetry metrics (a-c): P/M obtained by both manual delineations (a,b) and the automatic method (c) by disease group. Each dot corresponds to a subject, color represents the acquisition protocol. Shape analysis: Compatibility between acquisition protocols in shape analysis: Volume of the automatically segmented brainstem. Shape analysis is based in the 3D model obtained from this segmentation. Each dot corresponds to a subject, color represents the acquisition protocol.


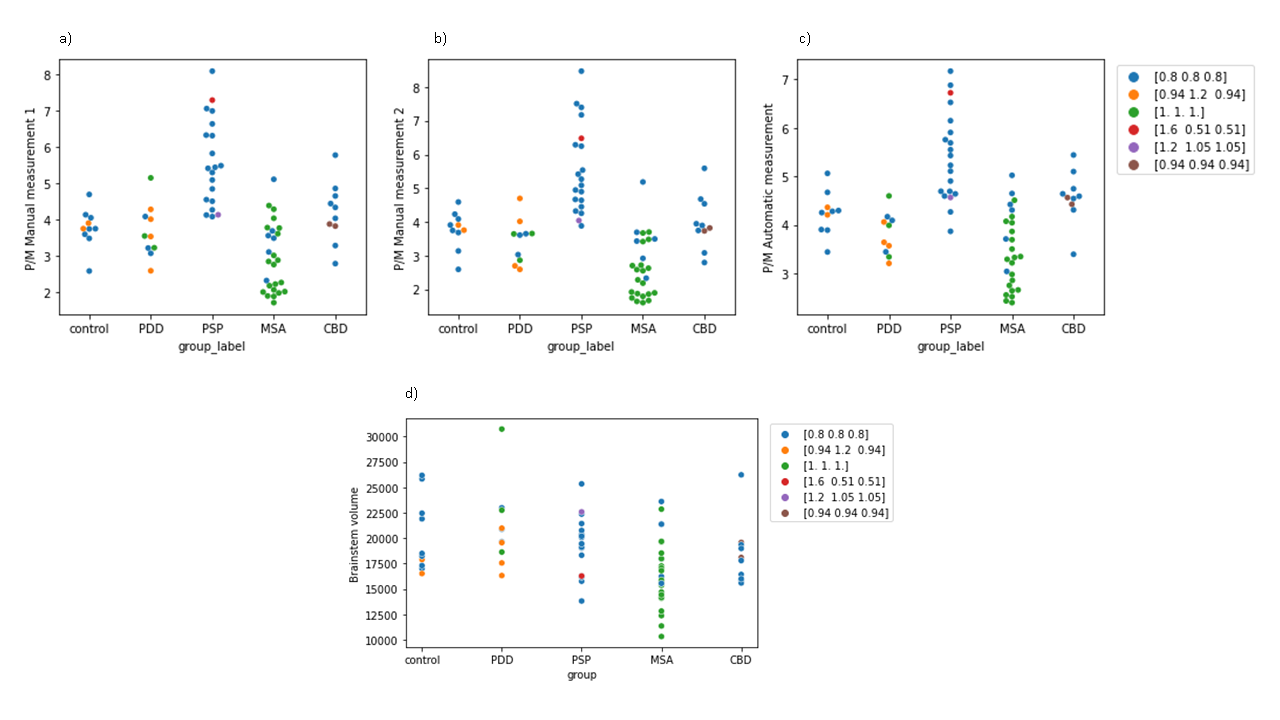


**Supplementary Fig.3 Brainstem automatic measures**

Sagittal slices of two subjects with the corresponding brainstem parcellation and the selected areas to calculate the P and M values automatically by calculating the sum of the areas of the total of the voxels of these regions.


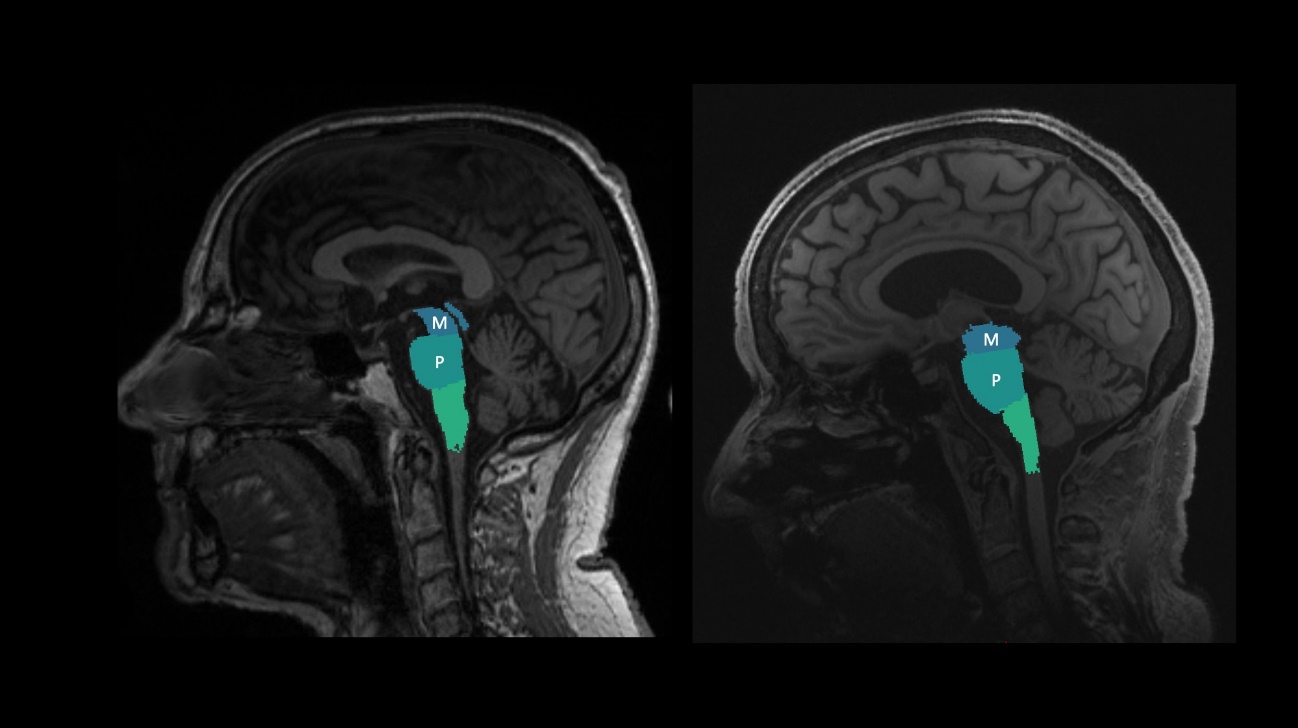


**Supplementary Fig.4 Manual and automatic segmentation of morphometric MRI measurements**

Midbrain and pons areas were measured manually at the midsagittal plane, as previously described[38]. Lines parallel to the mammillary–posterior commissural plane at the rostral and caudal pontine border were used to determine the midbrain and pons areas. a) shows representative manual planimetry measures in a subject with PD and b) in a subject with PSP.


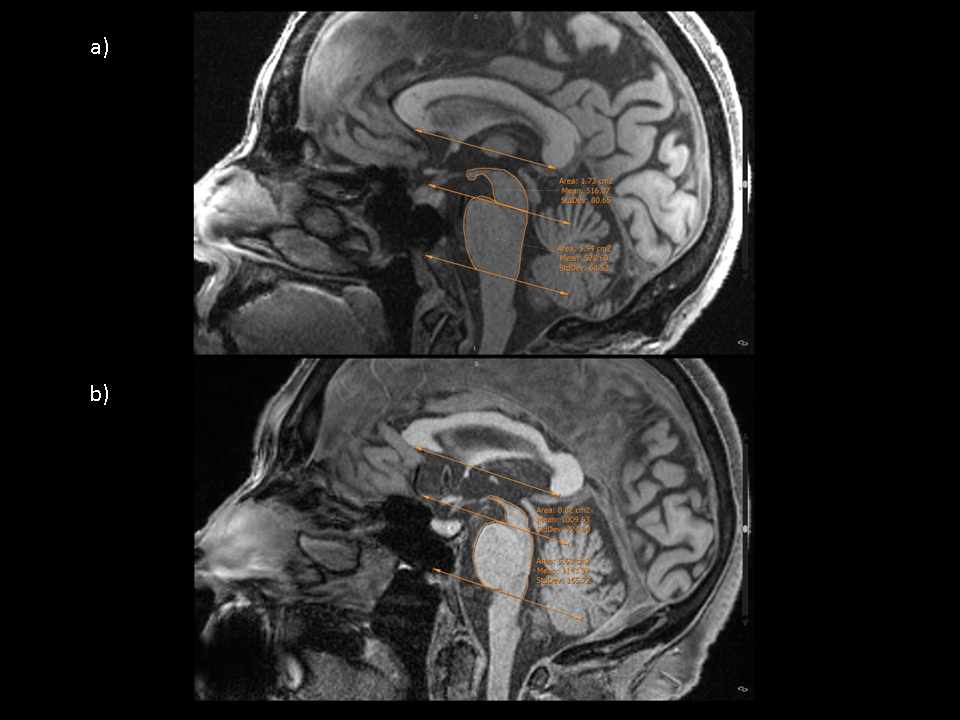


**Supplementary Fig. 5 Mean distance between the average shape of each pair of groups, non-significant results**

Warm to cold colormap showing in blue areas where the first group is narrower than the second group, and red for the opposite case. The vertical barcode represents the distance between groups in millimetres, being 4.3mm the maximum distance found between two groups. PSP and MSA showed the most extensive average atrophy with respect to the other groups. In the comparison between PSP and MSA, the first presented greater atrophy in the anterior and posterior midbrain and central pons regions and the second, greater atrophy in the lateral midbrain and in the middle cerebellar peduncles. CBD presented a similar shape to PSP, but to a lesser extent*,* when compared to PD and CS. CBD presented increased width of the pons area with respect to MSA. PD presented on average less atrophy of the brainstem shape when compared to other parkinsonisms, except in specific areas: lateral midbrain and pons in the PSP and CBD comparison and small regions in the midbrain in comparison with MSA. In addition, the average brainstem of the PD group showed smaller shapes in comparison with the average control.


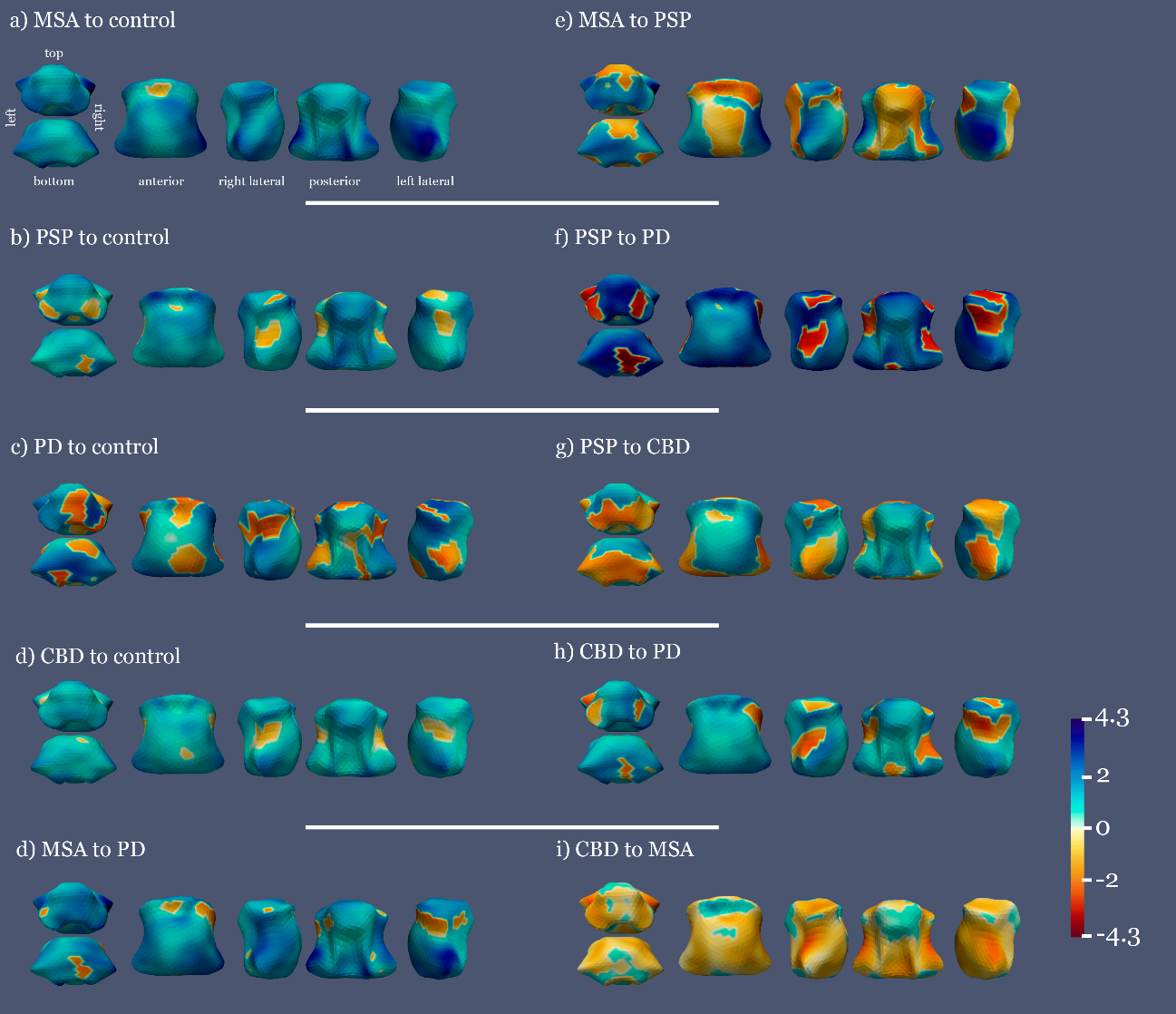


**Supplementary Fig. 6 Parcellation of the brainstem for the corresponding shape analysis.**

Sagittal (a) and coronal (b) sections from a subject selected for illustrative purposes in which the acquired T1w, the parcellation of the brainstem and the mesh used for the corresponding shape analysis are shown.


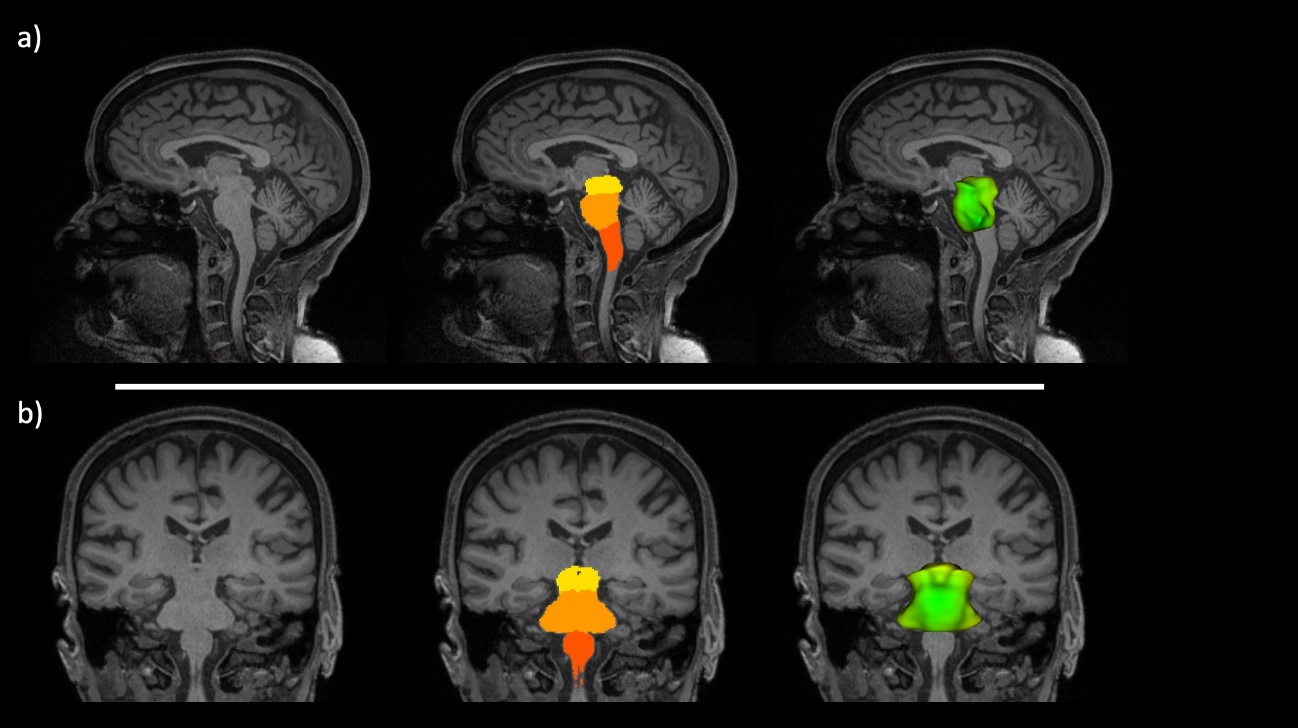


**Supplementary table 1. Details on the number of acquisitions with each protocol.**

|  | 0.8x0.8x0.8 | 0.94x0.94x0.94 | 1x1x1 | 0.94x1.2x0.94 | 1.2x1.05x1.05 | 1.6x0.51x0.51 |
| --- | --- | --- | --- | --- | --- | --- |
| Control | 8 |  |  | 2 |  |  |
| PDD | 3 |  | 3 | 4 |  |  |
| PSP | 18 |  |  |  | 1 | 1 |
| MSA | 6 |  | 20 |  |  |  |
| CBD | 8 | 2 |  |  |  |  |

**Supplementary table 2. Comparison of CSF and automatic radiological biomarkers across the different study groups covariated by age.**

| **CSF** | **PSP**  **(n =20)** | **CBD**  **(n =14)** | **MSA**  **(n =26)** | **PD**  **(n =10)** | **CS**  **(n =12)** | **p-value*** |
| --- | --- | --- | --- | --- | --- | --- |
| **NfL** | 1897.5  [1554.3 - 2441.2] | 2187.2  [1701.6 - 2718.3] | 2851.2  [1940.5 - 4278.7] | 815.7  [697.4 - 1108.2] | 598.6  [434.4 - 815.5] | **All groups**: <0.001; **PSP+MSA+CBD / PD+CS**: <0.001; PSP/CBD: 0.519; PSP/MSA: 0.269; **PSP/PD**: 0.001; **PSP/CS**: <0.001; CBD/MSA: 0.697; **CBD/PD**: <0.001; **CBD/CS**: <0.001; **MSA/PD**: <0.001; **MSA/CS**: <0.001; PD/CS: 0.115 |
| **MRI** | **PSP**  **(n =20 )** | **CBD**  **(n =10 )** | **MSA**  **(n =26 )** | **PD**  **(n =10 )** | **CS**  **(n =10 )** | **p-value*** |
| **M_A_ (cm^2^)** | 0.87  [0.79-1.04] | 1.01  [0.9-1.1] | 1.18  [1.04-1.29] | 1.28  [1.16-1.39] | 1.15  [1.1-1.22] | **All groups**: <0.001; PSP/CBD: 0.494; **PSP/MSA**: 0.014; **PSP/PD**: <0.001; **PSP/CS**: 0.005; CBD/MSA: 0.335; **CBD/PD**: <0.001; **CBD/CS**: 0.018; **MSA/PD**: 0.003; MSA/CS: 0.378; PD/CS: 0.060 |
| **P_A_**  **(cm^2^)** | 4.72  [4.41-5.19] | 4.55  [4.08-4.87] | 3.87  [3.41-4.42] | 4.81  [4.5-5.1] | 4.58  [4.4-5.62] | **All groups**: 0.003; PSP/CBD: 0.292; **PSP/MSA**: 0.003; PSP/PD: 0.890; PSP/CS: 0.890; CBD/MSA: 0.271; CBD/PD: 0.183; CBD/CS: 0.198; **MSA/PD**: 0.003; **MSA/CS**: 0.033; PD/CS: 0.890 |
| **PM_A_ (cm^2^)** | 5.33  [4.67-6.03] | 4.58  [4.43-4.75] | 3.34  [2.75-4.08] | 3.82  [3.45-4.09] | 4.27  [3.91-4.36] | **All groups**: <0.001; **PSP/CBD**: 0.039; **PSP/MSA**: <0.001; **PSP/PD**: <0.001; **PSP/CS**: <0.001; **CBD/MSA**: 0.020; **CBD/PD**: 0.005; CBD/CS: 0.191; MSA/PD: 0.99; MSA/CS: 0.191; PD/CS: 0.096 |

Quantitative variables are presented as median [IQR].

*p-values results obtained with ANCOVA with age as covariate. Results are presented FDR-corrected.

Statistically significant differences between groups are marked in **bold**.

|  | **PSP**  **(n =20 )** | **CBD**  **(n =10 )** | **MSA**  **(n =26 )** | **PD**  **(n =10 )** | **CS**  **(n =10 )** | **p-value*** |
| --- | --- | --- | --- | --- | --- | --- |
| **M_A_ (cm^2^)** | 0.99  [0.81-1.15] | 1.26  [1.17-1.33] | 1.70  [1.51- 1.80] | 1.59  [1.40-2.03] | 1.45  [1.39 - 1.70] | **All groups**: <0.001; **PSP/CBD**: 0.048; **PSP/MSA**: <0.001; **PSP/PD**: <0.001; **PSP/CS**: <0.001; **CBD/MSA**: 0.029; **CBD/PD**: 0.013; **CBD/CS**: 0.013; MSA/PD: 0.240; MSA/CS: 0.868; PD/CS: 0.516 |
| **P_A_**  **(cm^2^)** | 5.26  [4.98- 5.83] | 4.98  [4.27-5.49] | 4.13  [3.26- 4.92] | 5.34  [5.26- 5.74] | 5.21  [5.00 - 6.38] | **All groups**: <0.001; PSP/CBD: 0.275; **PSP/MSA**: 0.003; PSP/PD: 0.629; PSP/CS: 0.880; CBD/MSA: 0.240; CBD/PD: 0.126; CBD/CS: 0.275; MSA/PD: 0.001; **MSA/CS**: 0.030; PD/CS: 0.674 |
| **PM_A_ (cm^2^)** | 5.18  [4.55– 6.38] | 3.86  [3.73–4.53] | 2.57  [1.87– 3.43] | 3.63  [2.86– 3.65] | 3.83  [3.68– 4.08] | **All groups**: <0.001; **PSP/CBD**: 0.001; **PSP/MSA**: <0.001; **PSP/PD**: <0.001; **PSP/CS**: <0.001; **CBD/MSA**: 0.011; CBD/PD: 0.157; CBD/CS: 0.683; MSA/PD: 0.240; **MSA/CS**: 0.014; PD/CS: 0.225 |

**Supplementary table 3. Comparison of manual (Manual 1) MRI biomarkers across the different study groups covarying for age**

*p-values results obtained with ANCOVA with age as covariate. Results are presented FDR-corrected.

Statistically significant differences between groups are marked in **bold**.

**Supplementary table 4. Inter-rater agreement and consistency coefficients for the different measures.**

There was excellent degree of inter-rater agreement and consistency for PM ratio between manual measurements (Manual 1 vs. Manual 2) and, manual and automatic measures (Manual 1 vs. automatic). For M_A_ and P_A_, there was also excellent reliability between manual measurements. Between automatic and manual measurements, the agreement and consistency ICCs were fair and good for M_A_ and, good and excellent for P_A_ measures, respectively.

| **Between Manual 1 and Manual 2 measures** | | | **Between Manual 1 and automatic measures** | | |
| --- | --- | --- | --- | --- | --- |
|  | **ICC of consistency** | **ICC of agreement** |  | **ICC of consistency** | **ICC of agreement** |
| **M_A_** | 0.935 | 0.933 | **M_A_** | 0.747 | 0.585 |
| **P_A_** | 0.975 | 0.918 | **P_A_** | 0.917 | 0.709 |
| **PM** | 0.948 | 0.941 | **PM** | 0.875 | 0.857 |

**Supplementary table 5. Significant associations between CSF and automatic MRI measures with clinical variables**

Multiple linear regression models were composed by a dependent variable (UPDRS, UMSARS, PSPRS, MoCa, MMSE or SEADL) plus one independent variable (NfL, pTau, automatic M_A_ or automatic P_A_) and, were adjusted by age.

| **Sample Group** | **Model** |  |
| --- | --- | --- |
| PSP | PSPRS  NfL  Age | β: 0.28, p-value: 0.01. Model adjusted R^2^: 0.48 |
| PSP | SEADL  NfL  Age | β: -0.86, p-value: 0.02. Model adjusted R^2^: 0.32 |
| PD+PSP+CBD* | UPDRS  NfL  Age | β: 0.60, p-value < 0.001. Model adjusted R2: 0.34 |
| PD+PSP+CBD+MSA | SEADL  NfL  Age | β: -0.35, p-value: 0.02. Model adjusted R2: 0.15 |
| PD+PSP+CBD* | UPDRS  M_A_  Age | β: -0.44, p-value: 0.03. Model adjusted R2: 0.34 |

*MSA patients were not assessed with the UPDRS.
